# Controlling Intracellular Machinery via Polymer Pen Lithography Molecular Patterning

**DOI:** 10.1021/acscentsci.2c00683

**Published:** 2022-08-29

**Authors:** Millicent Lin, Brian Meckes, Chaojian Chen, Michelle H. Teplensky, Chad A. Mirkin

**Affiliations:** † Department of Biomedical Engineering, Northwestern University, 2145 Sheridan Road, Evanston, Illinois 60208, United States; ‡ International Institute for Nanotechnology, 2145 Sheridan Road, Evanston, Illinois 60208, United States; § Department of Chemistry, Northwestern University, 2145 Sheridan Road, Evanston, Illinois 60208, United States

## Abstract

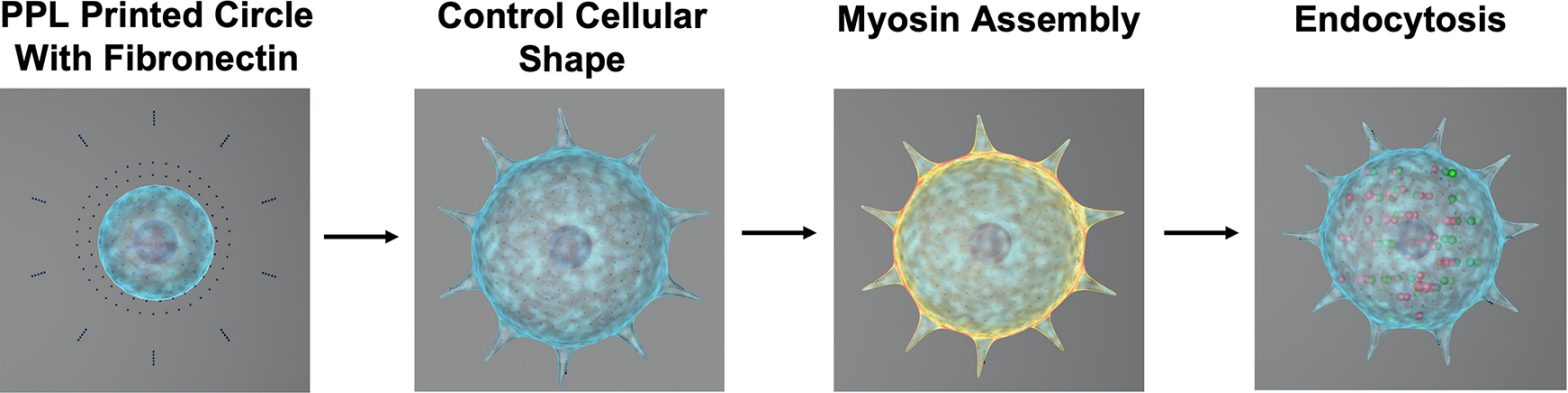

The plasma membrane and the actomyosin cytoskeleton play key roles in controlling how cells sense and interact with their surrounding environment. Myosin, a force-generating actin network-associated protein, is a major regulator of plasma membrane tension, which helps control endocytosis. Despite the important link between plasma membranes and actomyosin (the actin–myosin complex), little is known about how the actomyosin arrangement regulates endocytosis. Here, nanoscopic ligand arrangements defined by polymer pen lithography (PPL) are used to control actomyosin contractility and examine cell uptake. Confocal microscopy, atomic force microscopy, and flow cytometry suggest that the cytoskeletal tension imposed by the nanoscopic ligand arrangement can actively regulate cellular uptake through clathrin- and caveolin-mediated pathways. Specifically, ligand arrangements that increase cytoskeletal tension tend to reduce the cellular uptakes of cholera toxin (CTX) and spherical nucleic acids (SNAs) by regulating endocytic budding and limiting the formation of clathrin- and caveolae-coated pits. Collectively, this work demonstrates how the cell endocytic fate is regulated by actomyosin mechanical forces, which can be tuned by subcellular cues defined by PPL.

## Abstract 2

This work shows how myosin contractility controlled by nanoscopic ligand arrangements defined by polymer pen lithography regulates cellular uptakes through caveolae- and clathrin-dependent pathways.

## Introduction

Cells recognize and adapt to the extracellular matrix (ECM) through focal adhesions (FAs), large protein assemblies that link the cellular cytoskeleton to ECM proteins.^1−3^ Critically, the organization of FAs (size, density, spacing, and shape) mediates actomyosin assembly to generate and regulate forces within cells.^2,4−8^ The modulation of these forces and the accompanying membrane deformation contribute significantly to the membrane and cytoskeletal tensions that are known to mediate intracellular trafficking.^7,9,10^ Specifically, plasma membrane rigidity acts as a physical barrier, hindering the formation of clathrin- or caveolae-coated vesicles.^10^ Furthermore, acute mechanical stresses generated by myosin contractility can lead to the ejection of caveolae-coated pits.^11,12^ Taken together, cell internalization (via clathrin- and caveolae-mediated pathways) can be dramatically reduced by the FA-directed forces generated both at the plasma membrane and in the actomyosin cytoskeleton.

Altered uptake mechanisms have been observed in the context of numerous diseases (notably, cancer,^13,14^ osteoarthritis,^15^ and fibrosis^16,17^), where modified ECM landscapes and increased cellular contractility are frequently observed.^14,18−20^ Such changes could significantly impact drug delivery,^21^ as therapeutic uptake often occurs through clathrin- and caveolae-mediated internalization pathways.^10,12,22,23^ Therefore, alterations in uptake mechanisms could reduce therapeutic activity in target cells and thus limit efficacy. The development of models that allow one to probe this phenomenon in a controlled fashion could open new avenues for identifying next-generation therapeutic targets. It is critical to understand how ECM-mediated changes in the organization of the plasma membrane, particularly the myosin components, directly influence endocytosis pathways.

While previous studies have discussed the role of myosin as a regulator of plasma membrane tension, little is known about how changes in actomyosin organization affect endocytic trafficking and cellular uptake.^7,12,13,18^ Recent data highlight the advantages of using ligand patterning techniques to understand cell–ECM interactions.^2,6,24−31^ In fact, micropatterning techniques have become essential tools for reconstituting an ECM environment to examine the role of mechanical and morphological cues on various cell behaviors.^6,8,32−35^ While most conventional techniques employ *microscale* features, it is understood that the *nanoscale* arrangement of ECM ligands may provide more powerful and precise control over cellular behavior.^32,36^ However, generating features at such a small scale over large areas is challenging.^8^ A high-throughput, large-area patterning technique capable of printing submicrometer ECM features would be advantageous for exploring actomyosin organization and its biological effects. In our previous work, polymer pen lithography (PPL), a massively parallel, maskless, and cantilever-free scanning probe lithography technique, was used to modulate the assembly of the actin cytoskeleton independent of the cell shape by controlling the arrangement of adhesion ligands at the submicrometer length scale.^37−42^ Indeed, PPL has proven to be a powerful tool for investigating membrane and cytoskeletal morphology-based parameters related to endocytosis.^37,41^

In this study, nanopatterned geometric features that direct FA formation are used to control and study the cellular endocytic machinery in a myosin contractility-dependent fashion. Specifically, we use PPL to generate arrays of submicrometer features of 16-mercaptohexadecanoic acid (MHA) and then fibronectin, a key constituent of the ECM that promotes cell adhesion; these patterns can be used to dictate cytoskeletal organization and contractility (*vide infra*). In general, FA arrangements that increase cellular contractility lead to decreased clathrin- and caveolae-mediated endocytosis. Specifically, changing the cell architecture through pattern cues affects the cellular expression of ρ-associated protein kinase and the subsequent endocytic activities. Taken together, this work shows how pattern design parameters at the nanoscale can be used to deliberately and systematically toggle cell endocytic machinery for a variety of purposes in biology, the life sciences, and biomedicine.

## Results and Discussion

### Generation of Circular Patterns with Unique Peripheral Fibronectin Arrangements by PPL

**Figure 1:**
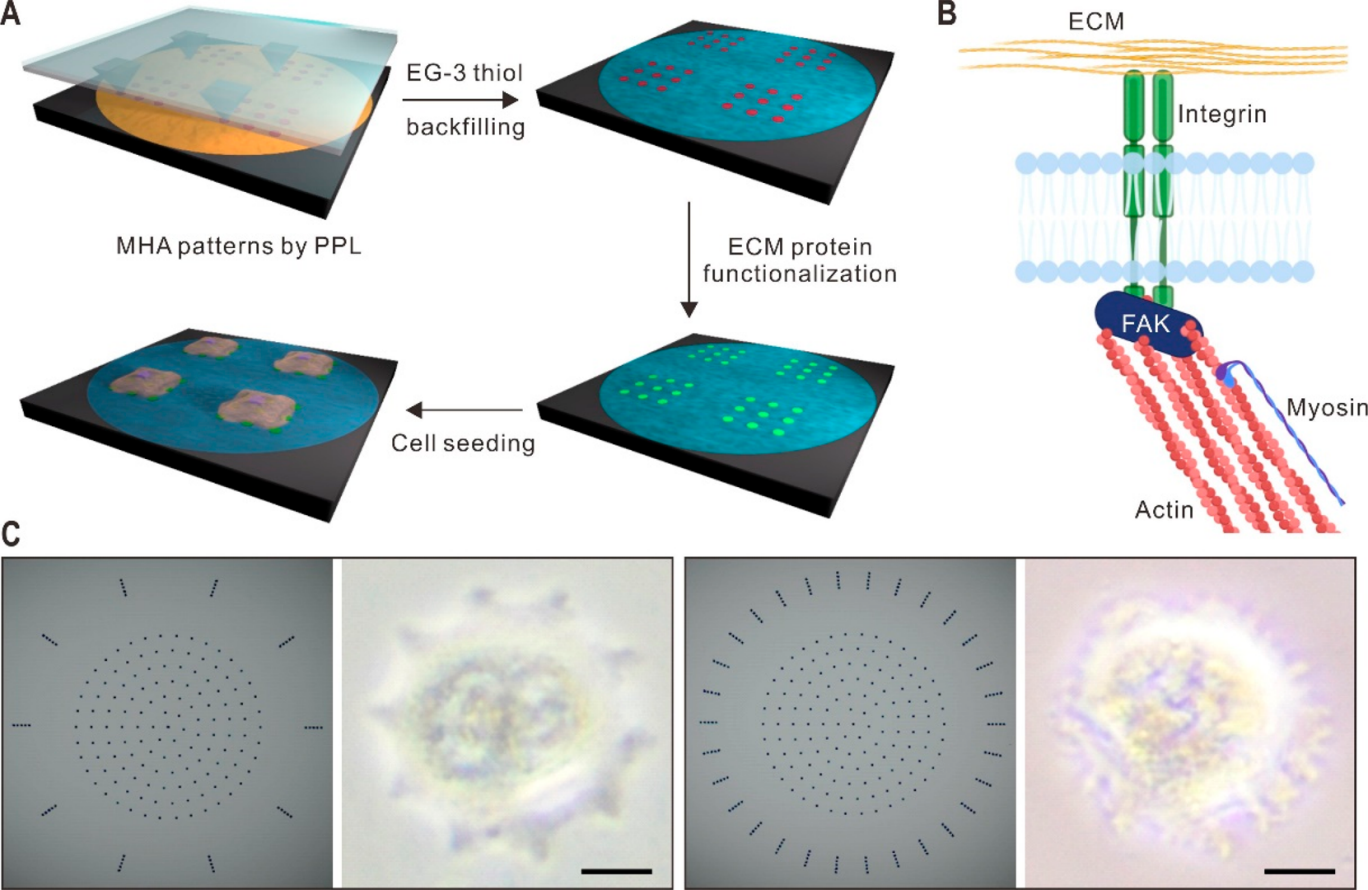
ECM protein patterns generated by PPL. (A) Schematic showing the key steps for generating molecular patterns: the deposition of MHA, backfilling the unoccupied area with a passivation layer, ECM protein immobilization on the patterned regions, and cell attachment to the molecular patterns. (B) Illustration of mechanical tensions exerted on the ECM by cells through focal adhesive contacts established by the myosin–actin interaction. FAK represents focal adhesion kinase. The figure was made using Biorender. (C) Representative pattern designs with different numbers of peripheral features (left) and optical micrographs of cell adhesion and cell morphology corresponding to the underlying pattern designs (right). Scale bars represent 10 μm.

To assess the effect of ligand arrangement on endocytosis, substrates presenting spatially defined nanoscale arrangements of fibronectin were synthesized using PPL following established methods (Figure 1A).^40,41^ For this study, circular patterns with different numbers of peripheral features, but the same geometry and spreading area, were designed and prepared to modulate cytoskeleton contractility and ultimately endocytosis. To generate these patterns, PPL arrays consisting of 10 000 tips were inked with MHA and loaded into a TERA-Fab PPL instrument. MHA was then deposited onto gold-coated glass slides in defined geometries. To validate the patterns, a portion of the patterned area was chemically etched and then visualized using an optical microscope (Figure S1). The bare gold was then backfilled by incubating the substrates in a bioinert polyethylene glycol (PEG) thiol, which prevented the nonspecific adsorption of proteins to the unpatterned regions (Figure 1A).^39^ After backfilling, following our reported protocol, the substrates were immersed in a fibronectin solution to facilitate cell adhesion to the MHA-defined patterns.^37^ The carboxylic acids in MHA will facilitate coordination with the proteins, allowing them to adsorb onto the surface. The fibronectin localized on the MHA patterns was visualized using immunofluorescence microscopy (Figure S2). Fibroblast cells were subsequently added to the patterned substrates.

### Actomyosin Architectures within Cells Seeded on the Patterns

**Figure 2:**
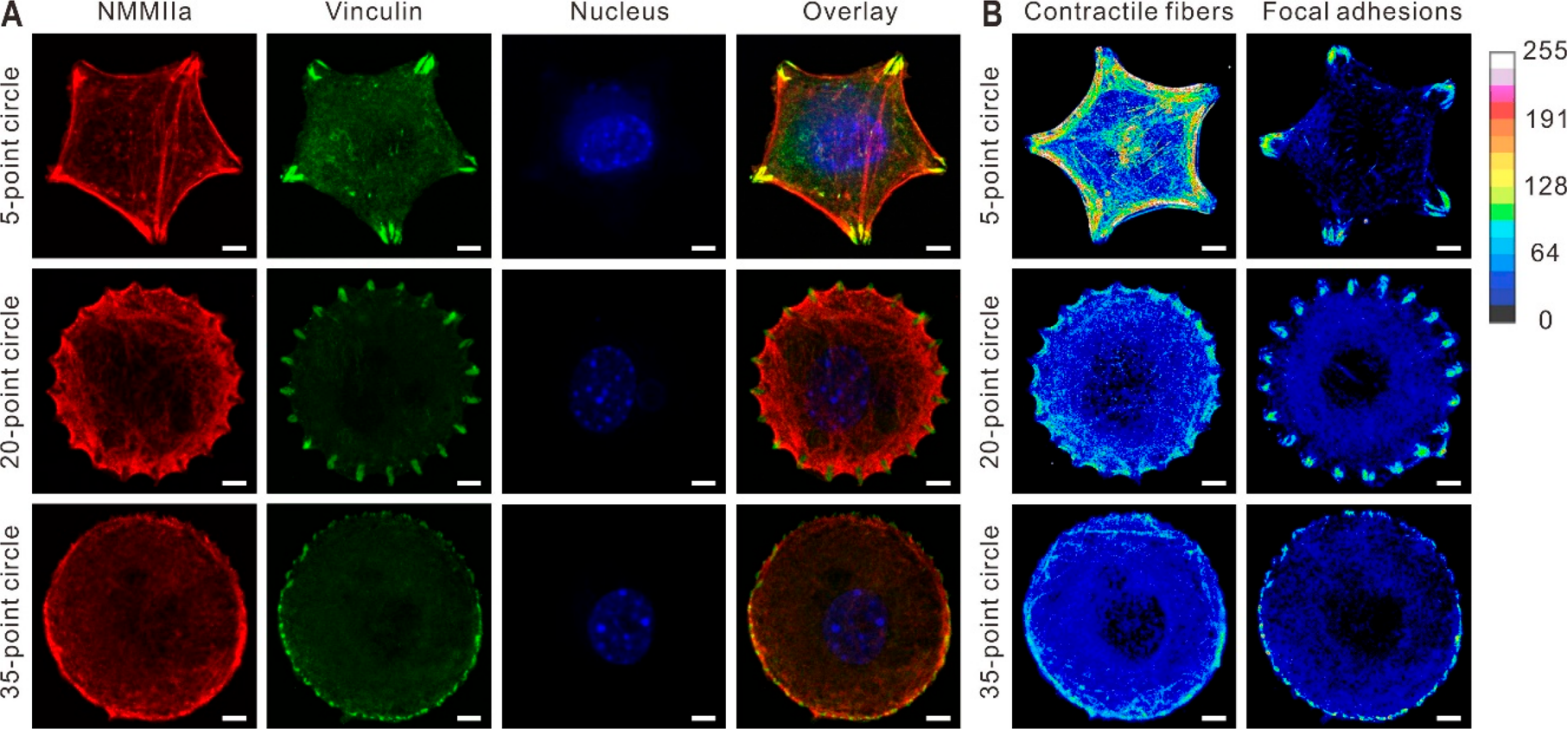
Cytoskeletal organization of fibroblast cells on patterns with different geometric cues. (A) Fluorescence micrographs of cells in 5-, 20-, and 35-point circle shapes stained for myosin IIA (NMMIIa), vinculin, and the nucleus. An overlay of these three micrographs is also shown. (B) Immunofluorescence heatmaps of the assembly of contractile fibers and focal adhesions generated by overlaying fluorescent micrographs of cells (*n* = 8) as a quantitative measure of the cell surface tension. Scale bars represent 5 μm.

To assess whether these patterns could be utilized to control cellular properties, including the organization and contractility of the actin cytoskeleton (Figure 1B), fibroblasts were cultured and immobilized on an array of patterns at 3000 cells/cm^2^ to enable single-cell attachment to individual patterns. The cells were incubated for 24 h and fully spread over the fibronectin patterns. Bright-field micrographs confirm cell adhesion to the fibronectin on the patterns and the adoption of the underlying pattern geometry (Figure 1C). After being stained with vinculin, a FA marker, and nonmuscle myosin IIa (NMMIIa), a primary force generator in the actin cytoskeleton, the cells were subsequently imaged using confocal microscopy. In this way, the changes in the actin cytoskeleton due to confinement of the FAs to different arrangements of surface-bound ligands could be examined. The immunofluorescence micrographs (Figure 2A) and heatmaps of the vinculin and NMMIIa localization (Figure 2B, generated by stacking images of multiple cells) indicate that more vinculin and NMMIIa are present at the cell peripheries directly corresponding to the underlying patterns, regardless of the number of peripheral features used.

**Figure 3:**
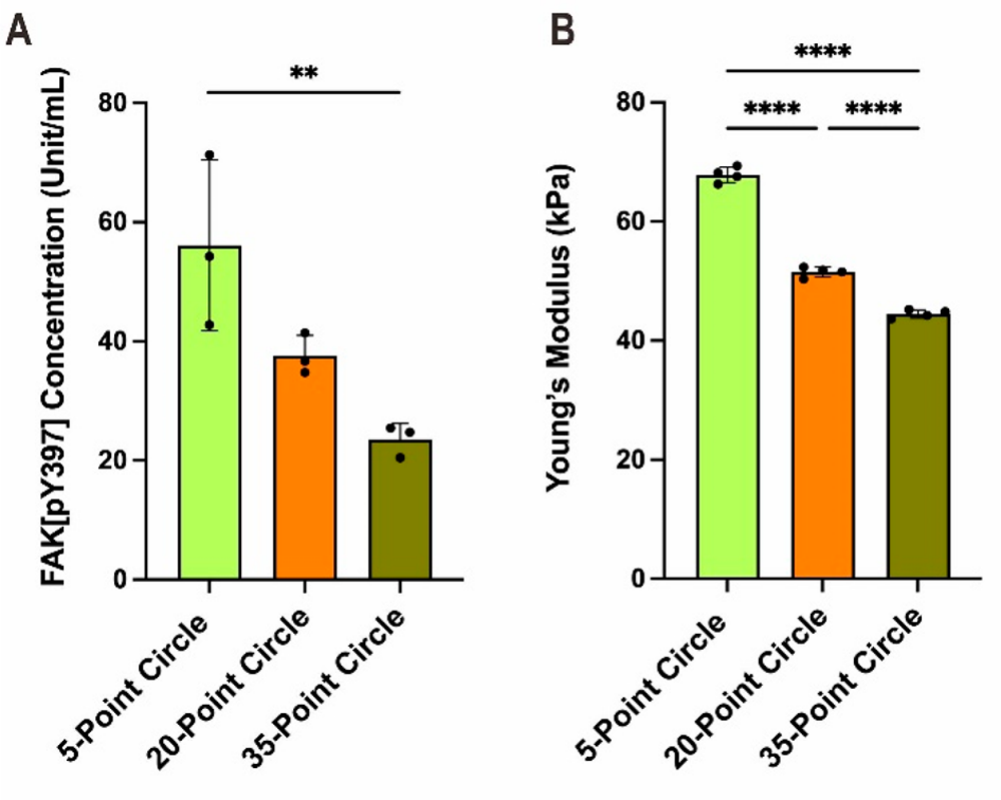
Mechanical differences in cells on patterns. (A) Quantitative analysis of the focal adhesion expression measured by ELISA. (B) Young’s modulus values of cells with different numbers of peripheral features as measured using AFM. Data are shown as mean ± SEM with *n* ≥ 3. Statistical analysis was performed using one-way ANOVA, followed by a multiple comparison test using Tukey *post hoc* analysis. **p < 0.01, ****p < 0.0001.

Importantly, for the cells on the patterns with increased spacing between peripheral features (5-point circle > 20-point circle > 35-point circle), the vinculin and NMMIIa were consistently more intense and more colocalized. The increased vinculin and NMMIIa intensities observed in the cells where the peripheral features were spaced further apart demonstrate that contractility increases with the ligand spacing, which is consistent with literature precedent^33,41^ (Figure 2). These data suggest that the cells on the 5-point circular patterns, which had the largest spacing between the fibronectin features, were the most contractile, while the cells on the 35-point circle pattern were the least contractile. In addition, the focal adhesion kinase (FAK) phosphorylation at tyrosine 397 (FAK[pY397]), an established response to increased actin contractility, was also examined.^3,6,7^ The total amount of FAK[pY397] was found to be elevated in the cells seeded on the 5-point circles compared to those seeded on the 35-point circles as measured by enzyme-linked immunoassays (ELISA) (Figures 3A and S3). In addition, we also found that patterned cells treated with blebbistatin,^33,43^ a specific inhibitor of NMMIIa, showed decreased total amounts of FAK[pY397] compared to patterned cells that were not treated with blebbistatin (Figure S4A). Additionally, cells cultured on 35-point circles with increasing aspect ratios displayed increased total amounts of FAK[pY397] (Figure S4B). These results are consistent with the role of myosin in influencing the number of focal adhesions, and features that promote contractility also favor focal adhesion formation.

The presence of actin and actomyosin organization greatly influences cell mechanical stiffness, so it was hypothesized that the cell stiffness would change depending on the spacing of the peripheral features.^3,7,9,13^ Therefore, cell stiffness was examined as a function of pattern geometry using atomic force microscopy (AFM). An analysis of the force curves generated by pressing on the centers of the cells using AFM probes indicted that stiffness increased as the number of peripheral features decreased (Figure 3B). The cells seeded on the 5-point circles had the highest Young’s modulus of ∼68 kPa (most stiff), while the cells seeded on the 35-point circles had the lowest Young’s modulus of ∼43 kPa (least stiff). These results support the above observation that cells with fewer peripheral features contain more vinculin and NMMIIa and have higher total amounts of FAK[pY397] and confirm that patterns that promoted greater actomyosin contractility also hosted cells with increased stiffness.

### Altering Endocytic Machinery by Controlling Actomyosin Contractility

**Figure 4:**
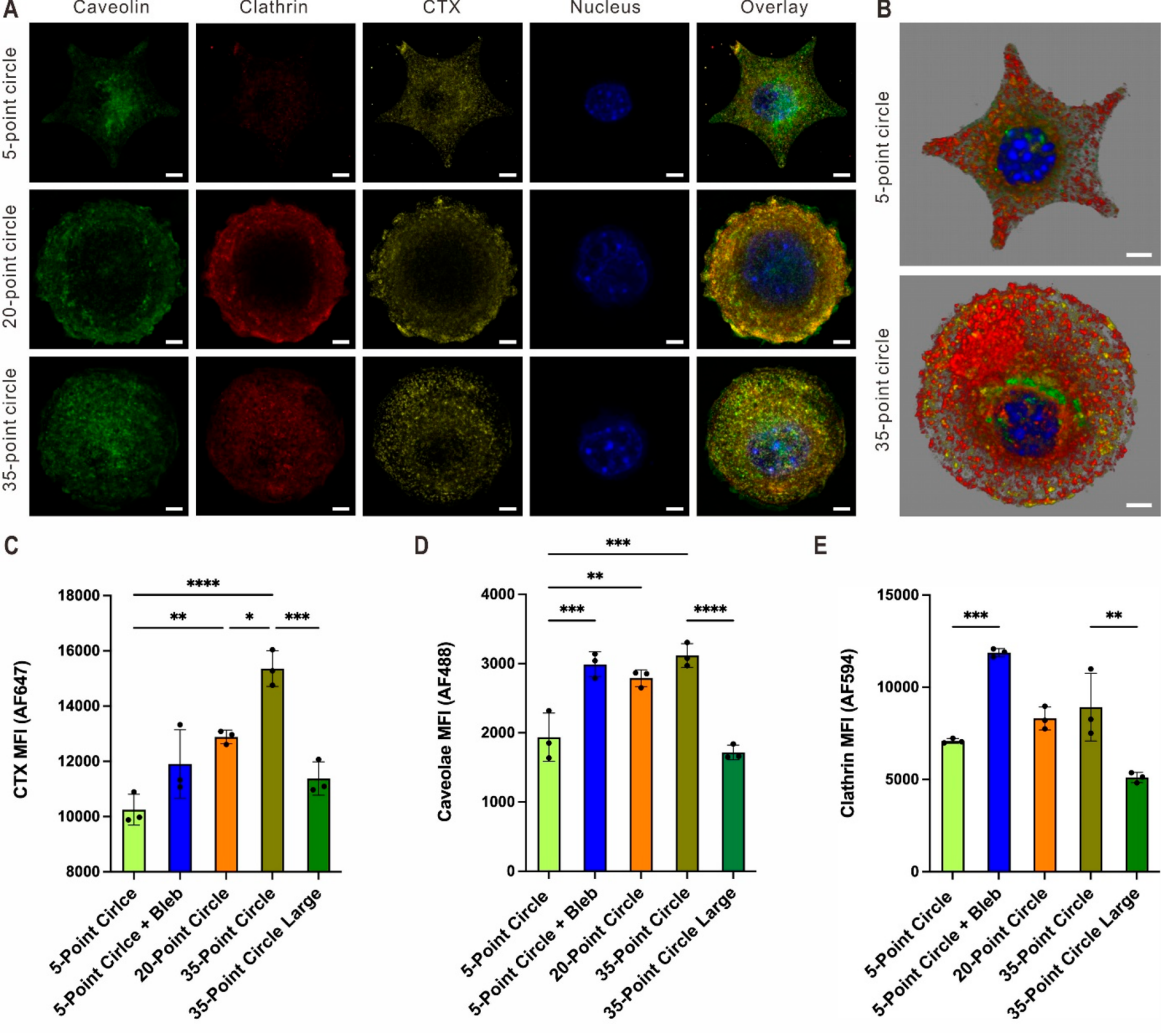
Cellular uptakes of probes via clathrin- and caveolae-specific pathways. (A) Confocal images showing caveolin (green), clathrin (red), cholera toxin (CTX) (yellow), and the nucleus (blue). The overlay images are in the rightmost column. (B) 3D confocal images of the internalization of CTXs (yellow) through clathrin- (red) and caveolae-mediated (green) pathways. (C–E) Flow cytometric analysis of the median fluorescent intensity (MFI) of cholera toxin uptake (AF 647) and caveolae- (AF 488) and clathrin-mediated (AF 594) endocytosis. The data are shown as mean ± SEM with *n* = 3. Statistical analysis was performed using one-way ANOVA, followed by multiple comparison tests using Sidak *post hoc* analysis. *p < 0.05, **p < 0.01, ***p < 0.001, ****p < 0.0001. Scale bars represent 5 μm.

It has been shown that the nanoscopic ligand arrangement can be used to control the stress fiber architecture and force generation. Next, we explored whether cell actomyosin tension could be used to regulate endocytosis. Specifically, the uptake of cholera toxin (CTX) protein complexes, which can enter cells through either clathrin- or caveolae-mediated endocytosis,^22,44−46^ were examined for cells seeded on the 5-, 20-, and 35-point circular patterns. The cells adherent on the patterns were incubated with CTX for 1 h, then the cellular uptake of CTX via each pathway were analyzed using confocal microscopy (Figure 4A and B). To gain quantitative information, flow cytometry was performed to measure changes in different uptake pathways following the fixation and removal of the cells from the substrate. Therefore, cell volume is not a parameter that will influence our measurements, since we are reporting the fluorescence intensity of the entire cell. Briefly, the cells were seeded on the patterns overnight to allow their cytoskeletons to reach homeostasis. Then, they were treated with fluorophore-labeled CTX for 1 h, and the CTX uptake was analyzed (Figure 4C). The data reveal that the uptake decreases as the contractility increases; the cells seeded on the 5-point circles (highest contractility) took up the least CTX, while those seeded on the 35-point circles (lowest contractility) took up the most CTX. To determine how the CTX uptake affected the cellular regulation of clathrin or caveolae vesicles, the fixed and permeabilized cells were treated with antibodies against clathrin and caveolae (Figures 4D and E). Interestingly, both clathrin and caveolin expression were reduced in the cells that exhibited increased contractility. Specifically, our data suggest the trend that clathrin and caveolae expression was lowest in the cells seeded on the 5-point circular patterns and greatest on those seeded on the 35-point circular patterns.

Subsequently, the cells seeded on the contractility-promoting 5-point circular patterns were treated with blebbistatin to partially negate geometry-dependent contractility within them. When blebbistatin was incubated with these cells overnight, CTX uptake and clathrin and caveolae levels increased (Figure 4C–E, respectively). Conversely, cells were seeded on 35-point circular patterns with larger spreading areas to increase their contractility. In particular, the diameter of the 35-point circle increased from 42 to 54 μm (a 65% increase in spreading area); at the same time, the spacing between the external features also increased. The cells on the larger patterns (54 μm) took up less CTX and showed reduced clathrin and caveolae content compared to those on the smaller diameter 35-point patterns (Figure 4C–E, respectively). Next, uptake with pHrodo green dextran was analyzed to investigate the connection between cell geometry and overall intracellular processing, which can reveal the relative amounts of the pH-sensitive conjugates that were taken up and encapsulated by phagocytosis or endocytosis vesicles. The data indicate strong correlations between the cell geometry and the intracellular fates of the probes; elevated fluorescent intensity was observed in cells seeded on 20-point and 35-point patterns (Figure S5). These results point to the fact that elevated contractility decreases endocytosis processes by downregulating clathrin and caveolae expression. The trend matches with the visual inspection of the confocal data and provides strong evidence that the material uptake and endocytic regulation can be altered through the direct manipulation of pattern geometry.

### ρ-Associated Protein Kinase (ROCK) Regulates Clathrin- and Caveolin-Mediated Endocytosis

**Figure 5:**
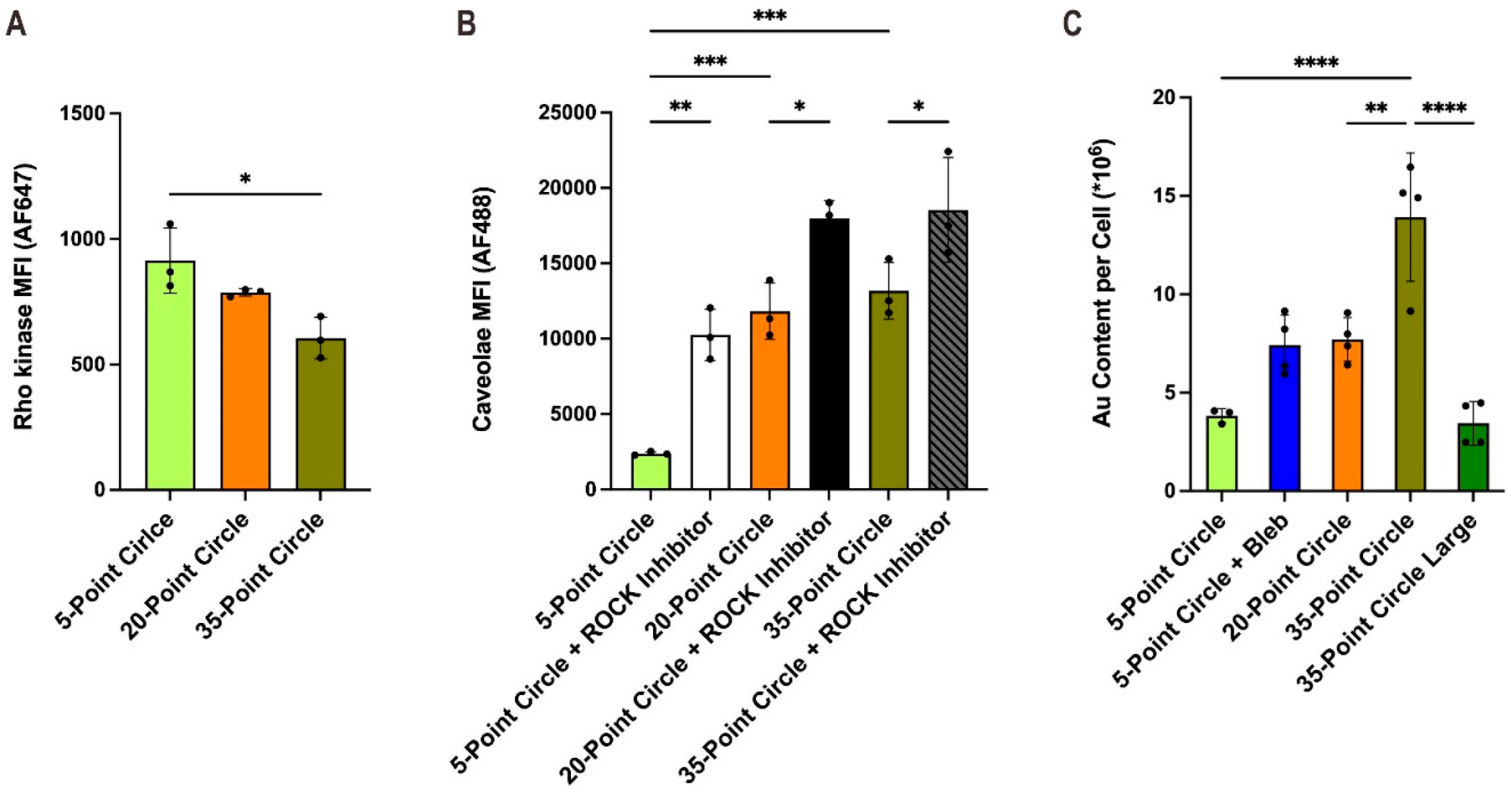
CTX and Au-cored SNA uptake experiments reveal that caveolae disassembly is highly associated with pattern-induced ρ-kinase expression and downstream myosin regulation. (A) Flow cytometric data shows that ROCK expression is elevated in high-contractile fibroblasts. (B) The addition of a ρ-kinase inhibitor promotes caveolae activity compared to that of the ρ-kinase inhibitor-absent groups. (C) Quantification of SNA uptake into cells on different patterns by ICP-MS. Statistical analysis was performed using one-way ANOVA, followed by multiple comparison tests using Tukey *post hoc* analysis for panel A and Sidak *post hoc* analysis for panels B and C. *p < 0.05, **p < 0.01, ***p < 0.001, ****p < 0.0001.

To assess the pathway by which clathrin and caveolae are reduced in the high-contractility systems, a messenger that translates actomyosin contractility into biochemical signaling cascades was examined. Specifically, the ROCK pathway, a well-established messenger of stress at FAs that plays a role in the disassembly of caveolae,^47−52^ was investigated (Figure 5A and B). To quantitatively investigate the interplay between ρ-kinase activity and endocytic behavior, particularly in the caveolae-dependent pathway, the uptake of CTX and its subsequent trafficking were further assessed. CTX uptake and caveolae expression (without and with the addition of a ρ-kinase inhibitor) in patterned cells followed the order 35-point circle > 20-point circle > 5-point circle, likely due to the decreased degree of cellular stress and membrane deformation with the cells with higher numbers of peripheral features (Figure 5A and B, *vide supra*). This ρ-kinase inhibitor y-27632 should decrease processing along the ROCK pathway and perhaps the ability to regulate endocytosis via contractility. In fact, the cells treated with ρ-kinase inhibitor were found to have greater caveolae activity than those that were not treated for the same number of peripheral features. Therefore, decreases in caveolae activity are correlated with increases in actin contractility.

### Influence of Cell Geometry on SNA Uptake

To further understand how the mechanical properties of the actin cytoskeleton may regulate nanomaterial-based therapeutics, the uptake of gold nanoparticle-cored spherical nucleic acids (SNAs) was investigated. SNAs typically consist of a spherical nanoparticle core, which serves as a template for a dense and highly oriented nucleic acid shell. Many different versions of SNAs have been used extensively in biomedicine, especially in sensing and biodetection, gene regulation, and immunotherapy.^53−55^ Importantly, SNAs have been found to enter cells via caveolae-mediated endocytosis pathways.^56^ Thus, cells on patterns with different numbers of features were treated with SNAs. Then, inductively coupled plasma mass spectrometry (ICP-MS) was performed on cell lysates to quantitatively evaluate the SNA uptake. Consistent with previous observations, the uptake of SNAs was downregulated in cells with elevated actin contractility, as dictated by the geometric cues (Figure 5C). To determine if this downregulation stemmed from actin contractility, the most contractile cells (those seeded on the 5-point circles) were treated with blebbistatin to inhibit NMMIIa. Consequently, increased accumulation of SNAs was observed in those blebbistatin-treated cells compared to those that were not treated with blebbistatin (Figure 5C). Moreover, the cells patterned on the 35-point circles showed threefold higher gold contents than those seeded on the 5-point patterns and twofold higher gold contents than those cells seeded on the 20-point circular patterns. The cells seeded on the larger-diameter 35-point circle patterns showed a significant decrease in uptake compared to those seeded on the smaller-diameter 35-point patterns, indicating that the identical pattern designs with increased diameters resulted in higher membrane stress and reduced probe uptakes.

## Conclusion

In summary, we have developed a way of using PPL-generated nanopatterns of ECM proteins to investigate the influence of such structures on chemical cargo uptake by fibroblast cells. This work shows how the endocytic process is coupled to the dynamic changes of plasma membrane components. Importantly, cell shape, controlled using patterned geometric cues on surfaces, strongly influences myosin assembly. Critically, the shape–cue trends promoted by the patterns mediated the organization of FAs and the actomyosin assembly that generated forces within the cells. The modulation of those forces, which led to subsequent membrane deformation, contributed significantly to the endocytic uptake mechanism. Specifically, regulating matrix stiffness through PPL-based pattern designs creates different myosin contractility profiles that alter mechano-sensitive signaling pathways, ultimately leading to differences in the numbers of clathrin- or caveolae-coated pits. This work also reveals how changing myosin-based focal adhesions using pattern-tuned mechanical tension and cytoskeletal reorganization can promote specific downstream signaling pathways and different cellular outcomes. Furthermore, this cell engineering approach enables one to study or mimic complex biological systems that may provide insights into new therapeutic approaches. Finally, it holds the capability to expand ECM libraries to other connective tissue diseases or cell types, and the high-throughput screening of cell–microenvironment interactions can significantly augment the current drug development processes.

## Supporting Information Available

Materials and methods, cell experiments, and supplementary data (PDF)
