# Supplementary material for: Controlling Intracellular Machinery via Polymer Pen Lithography Molecular Patterning
Source: ACS Cent Sci. 2022 Aug 29;8(9):1282–9. doi: 10.1021/acscentsci.2c00683 (PMC9523772; doi:10.1021/acscentsci.2c00683)
Supplement: Supplementary file 1 — oc2c00683_si_001.pdf [file oc2c00683_si_001.pdf]

Supporting information

Controlling Intracellular Machinery via Polymer Pen Lithography Molecular Patterning

Millicent Lin,[1,2]† Brian Meckes,[2,3]† Chaojian Chen,[2,3] Michelle H. Teplensky,[2,3] and Chad A. Mirkin[1,2,3]*
[1] Department of Biomedical Engineering, Northwestern University, 2145 Sheridan Road, Evanston, Illinois 60208, United States
[2] International Institute for Nanotechnology, 2145 Sheridan Road, Evanston, Illinois 60208, United States
[3] Department of Chemistry, Northwestern University, 2145 Sheridan Road, Evanston, Illinois 60208, United States
† These authors contributed equally to this work.
*Email: chadnano@northwestern.edu (C.A.M.)

Keywords: endocytosis, nanopatterning, polymer pen lithography, actomyosin assembly, cell membrane tension

Methods

Materials. All reagents and chemicals were purchased from commercial resources unless otherwise stated. Cell culture reagents, Dulbecco's modified eagle medium (DMEM) and P-FAK were purchased from Fisher Scientific and Gibco. NIH/3T3 embryonic fibroblast cells were obtained from ATCC. Fibroblast cells were cultured at 37 ° C with 5% CO_2 in DMEM supplemented with 10% fetal bovine serum (ATLANTA biologicals) and 1% pen strep (Gibco). Blebbistatin was purchased from Calbiochem. Y-27632 was purchased from R&D systems. The Exosome ELISA complete kit was purchased from System Biosciences. Protease inhibitor cocktail, 11-mercaptoundecyl)hexa(ethylene glycol), 16 mercaptohexadecanoic acid, protease inhibitor, donkey serum, and anti-fibronectin antibodies were purchased from Sigma. Fixation and permeabilization solution and BD perm wash buffer were purchased from BD Cell Analysis. Cholera toxin, exosome isolation reagents, anti-phalloidin, cell extraction buffer, and FAK pY397 ELISA kits were purchased from Life Technologies. Human fibronectin was purchased from EMD Millipore. Anti-ROCK antibody, mouse anti-vinculin, rabbit anti-myosin IIA, mouse anti-clathrin, rabbit anti-caveolin, and rabbit anti-FAK were purchased from Abcam. Prolong gold antifade mountant with 4',6-diamidino-2-phenylindole (DAPI) and live/dead fixable blue dead cell stain kits were purchased from Thermo Fisher. BCA protein assay kits were purchased from Thermo Scientific Pierce Biotech. Phenylmethylsulfonyl fluoride (PMSF) was obtained from Cell Signaling Technology. Glass slides were purchased from Ted Pella.

Safety Statement. No unexpected or unusually high safety hazards were encountered.

Polymer pen array and substrate fabrication. Polymer pen arrays were fabricated by mixing polydimethylsiloxane (PDMS) precursor at a ratio of 10:1 with the curing agent and pouring it on the master pen arrays (pen to pen distance = 150 μm).[1] Glass slides were rinsed with isopropanol and cleaned with O_2 plasma for 3 min. The glass slides were placed on the PDMS and left in the oven at 80 °C for 24 hr. The glass slides were separated from the master pen arrays with a razor blade and the surfaces were cleaned thoroughly with a N_2 stream. 1.9 x 1.9 cm^2, 0.5 mm thick glass slides (Ted Pella) were rinsed in ethanol and sonicated for 30 min. They were then dried and deposited with 5 nm of Ti and 35 nm of Au using an electron-beam evaporator (Lesker).

Patterning MHA features. The PDMS pen array was plasma-treated for 2 min at 10 W to make the surface hydrophilic. The tip array was inked via drop-casting 60 μL of 10 mM of mercaptohexadecanoic acid in ethanol onto the array. Au-coated glass substrates were then placed on the PPL system (TERA-Fab M series, TERA-print) secured to a stainless-steel chip using carbon tape. The pen was lowered until it was 100 μm above the substrate, then the tips were monitored using an optical microscope as the z-stage moved towards the substrates in small increments. Contact between the tips of the array and the substrate was further confirmed by visible deformation of the polymeric tips. After alignment, specific patterns were designed and executed by the PPL software (Figure S1). To confirm pattern transfer, some patterned substrates were etched for visualization by placing the substrates face-up in a well plate filled with freshly prepared Au etchant for ~5 min. The etched substrates were rinsed with H_2O and dried before observation (Figure S2).

Deposition of fibronectin on MHA patterns. Unetched glass substrates were patterned with MHA ink (**Patterning MHA features**). The substrates were backfilled with 1 mM solution of (1-mercapto-11-undecyl)hexa(ethylene glycol) in ethanol for 1 hr to prevent non-specific binding of cells or proteins. Prior to incubation of fibronectin solution, the substrates were thoroughly rinsed with ethanol and H_2O. A six-well plate was then filled with 50 μg/mL human plasma fibronectin (Millipore) in 1× phosphate buffered saline (PBS; pH 7.0) and placed on a shaker stirring (100 r.p.m.) overnight at 4 °C. The sample was then rinsed with 1× PBS after fibronectin incubation. The pattern quality was examined using immunofluorescence microscopy by incubating the patterned substrates with primary antibody (1:100, human anti-fibronectin produced in rabbit, Sigma-Aldrich F3648) diluted in 1× PBS overnight followed by incubation of fluorescently labeled secondary antibody (1:250, goat anti-rabbit Alexa Fluor 488, Abcam ab150077) in 1× PBS (Figure S3).

Seeding of NIH3T3s. NIH3T3s were seeded on the patterned substrates that varied in their numbers of peripheral features at a density of 3,000 cells/cm^2. Fibroblasts cells were cultured on patterned substrates overnight.

Immunofluorescence. After overnight incubation, the NIH3T3 cells on patterned substrates were fixed with 4% formaldehyde for 10 min and subsequently permeabilized with 0.1% Triton X-100 for 15 min followed by three rounds of washing. They were then blocked with 1% BSA diluted in 1× PBS for 1 hr. The substrates were then labeled with rabbit anti-myosin IIa (1:200, rabbit polyclonal to non-muscle Myosin IIA, Abcam ab75590) and mouse anti-vinculin (1:200, mouse monoclonal to Vinculin, Abcam ab130007) followed by secondary antibody staining (1:1000, goat

anti-rabbit Alexa Fluor 488, Invitrogen A32731. 1:1000, goat anti-mouse Alexa Fluor 555). The substrates were then mounted on a coverslip using Prolong Gold Antifade mountant with DAPI (Invitrogen) for visual observation using immunofluorescence microscopy.

In uptake experiments and qualitative studies of different endosomal pathways, cells were incubated with fluorescently labeled cholera toxin (CTX) conjugates for 1 hr. After washing the substrates with 1× PBS, the surfaces of the substrates were fixed with 4% formaldehyde and incubated with blocking solution (0.1% Triton X-100 and 1% BSA diluted in 1× PBS) for 1 hr. The fixed cells then were labeled with rabbit anti-caveolin (1:200, rabbit polyclonal to caveolin-1, Abcam ab2910) and mouse anti-clathrin (1:200, mouse monoclonal to clathrin heavy chain, Abcam ab2731) diluted in 1× PBS overnight at 4 ^{o}C on a shaker. Secondary antibody labeling was performed using either Alexa-488 or Alexa-555 goat anti-rabbit or anti-mouse (Invitrogen). The substrates were further mounted onto the coverslips using the same mounting procedure as described above. Immunofluorescence confocal microscopy was performed using a Leica SP8.

Focal adhesion kinase phosphorylation assay. Prior to performing focal adhesion kinase phosphorylation assays, cells were trypsinized, neutralized with cell culture media and centrifuged at 500 x g for 5 min at 4 ^{o}C. Cell pellets were incubated with cold 1× cell extraction buffer on ice for 20 min followed by centrifugation at 18,000 x g for 20 mins at 4 ^{o}C. The supernatants were collected and transferred into clean tubes, and the pellets were discarded. The total protein concentration in the extract was quantified using a Pierce BCA protein assay kit. The phosphorylation of FAK at Y397 was assessed using an ELISA assay (Invitrogen) performed according to the manufacturer's protocol.

AFM analysis. After the cells were fully attached and spread on the pattern areas, their mechanical properties were assessed. A Bruker High Performance Bioscope Resolve with an Asyst liquid cantilever probe (nominal spring constant of ~0.6 N/m) was used for all experiments. The true spring constant was determined using the thermal tune. All mechanical measurements were carried out in a liquid environment. Force spectroscopy was acquired at multiple locations across multiple cells (n=8) to get an estimate of the average modulus. The data was fit in Hertzian.

Au SNA uptake studies. After overnight incubation was complete, the cells were fully attached and spread. The cells then were incubated with 10 nM Au SNAs synthesized using a previously published protocol for 1 hr.[2] The glass substrates were washed with 1× PBS and the adherent cells were trypsinized and neutralized with cell culture media. Three-fourths of the sample volume was transferred to a 15-mL Falcon tube and centrifuged at 1,500 rpm at 4 ^{o}C for 5 min. The supernatants were removed while keeping the cell pellets intact. One-fourth of the sample volume was used to obtain duplicate cell counts using the Countess automated cell counter. Cell lysates were normalized by collecting the cells from the substrates, counting them, and then measuring lysates from equal numbers of cells. One-hundred and twenty μL of HCl and HNO_3 were added to the samples in the 15-mL Falcon tubes. Cells were left in acid for 30 min at room temperature until the samples turned clear yellow. In the meantime, a blank containing 1 mL of both HCl and HNO_3 and 50 mL of H_2O was made along with a 200 ppb gold calibration standard. The samples were diluted in 2 % HNO_3 HCl (> 69%, Thermo Fisher Scientific) and 2 % HCl (>34 %, Thermo Fisher Scientific) (v/v) solution, and the gold concentrations were determined using inductively coupled plasma mass spectroscopy (iCAP Q ICP-MS from Thermo Fisher Scientific).

Flow cytometry. After overnight cell culture, the cells were incubated with cholera toxin conjugates for 1 hr. After washing the substrates with 1× PBS, the cells were scraped and transferred to flow tubes. The cells were fixed with BD Cytofix/Cytoperm fixation and permeabilization solution (BD Biosciences, 554722) that contains 4.2% formaldehyde for 20 min at 4 °C, followed by washing and a 30-min incubation of 0.5 μL of primary antibodies per each tube (anti-ROCK kinase, Santa Cruz Biotechnology sc-17794AF647; rabbit anti-caveolin, Abcam ab2910; mouse anti-clathrin antibodies, Abcam ab2731). The supernatants were removed after washing and spun down to prepare them for the addition of 0.5 μL of secondary antibodies in each tube (goat anti-rabbit Alexa fluor 488, Invitrogen a32731; goat anti-mouse Alexa fluor 555, Invitrogen 32727). The cells were then incubated with secondary antibodies for 30 min at 4 °C in the dark, followed by washing, aspirating, and resuspending in 1× PBS. BD FACsymphony A3 cell analyzer was used to run the samples, and the data were analyzed using FlowJo.

Inhibition assays. Inhibitors such as Blebbistatin (Myosin IIa inhibitor; 10 μM) or Y-27632 (ROCK inhibitor; 2 μM) were added to the cell culture media and incubated for 24 hr. The substrates were immediately rinsed with PBS followed by incubation of cholera toxin conjugates for 1 hr. After washing the substrates with 1× PBS, the cells were collected using TrypLE solution (Thermo Fisher) and neutralized with cell culture media before transferring to flow tubes. The contents were transferred to flow tubes and spun at 1,500 rpm for 5 min. After the removal of media, 0.5 μL of live and dead stain solution was added to each tube and the sample were incubated for 20 min at 4 °C in the dark. The contents then were incubated with fixation and permeabilization buffer for 20 min at 4 °C in the dark. BD Perm/Wash (BD Bioscience, 51-2091KZ) diluted in H_2O was added directly to the vials followed by centrifugation and removal of the supernatants. 0.5 μL

of primary antibodies (rabbit anti-caveolin, Abcam ab2910; mouse anti-clathrin antibodies, Abcam ab2731) were added to the contents and incubated for 30 min at 4 ^{o}C followed by secondary antibody staining (goat anti-rabbit Alexa fluor 488, Invitrogen a32731, goat anti-mouse Alexa fluor 555, Invitrogen 32727) for 30 min at 4 ^{o}C. The cells were washed, aspirated, and resuspended in 1× PBS. BD FACsymphony A3 cell analyzer was used to run the samples, and the data were analyzed using FlowJo.

pHrodo green dextran assays. The pHrodo green dextran method was used to determine the amount of material internalized during endocytosis. The endosomal or lysosomal lumen is more acidic compared to the cytoplasm as the result of the ATP pumps present in endosomal and lysosomal membranes. Therefore, the acidic environments of both the endosomes and lysosomes trigger the green fluorescence signal from the dextran conjugate at 509 nm. After overnight cell attachment, the cells were fed with a 0.1 % solution of dye by volume. After a 4 hr treatment, the cells were washed once with PBS, then 300 μL of Tryple solution was added to the cells. After cell detachment, 300 μL of media was added to each well, and the contents were transferred to flow tubes. Cells were spin down at 1,500 rpm for 5 min and washed with 1× PBS. The media was then removed and replaced with solution from a Live/Dead Fixable Blue Dead Cell Stain kit (Thermo Fisher), followed by a 20-min incubation in the dark. The cells were aspirated, and PBS was added to get rid of excess live-dead stain. The contents were run on the flow instrument immediately after addition and incubation of fixation buffer.

Statistical Analysis.

GraphPad PRISM 9 (GraphPad Software, Inc.) was used for data analysis. The difference between two groups was compared using an unpaired *t*-test. Quantitative data assessing more than two groups were analyzed using one-way ANOVA with Tukey *post hoc* or Sidak *post hoc* analysis. P value styles were as follows: $p>0.05$ (ns), $p<0.05$ (*), $p<0.01$ (**), $p<0.001$ (***), $p<0.0001$ (****). The significance threshold was set to $p=0.05$.

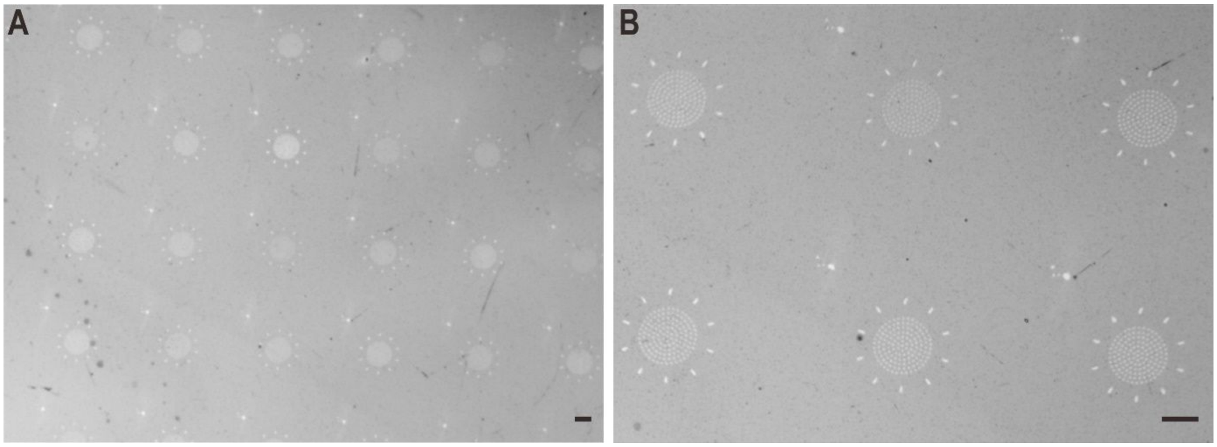

Figure S1. Micrographs of Au nanopatterns templated by MHA features after etching to confirm the successful preparation of molecular patterns generated by PPL. Scale bars: 20 μm.

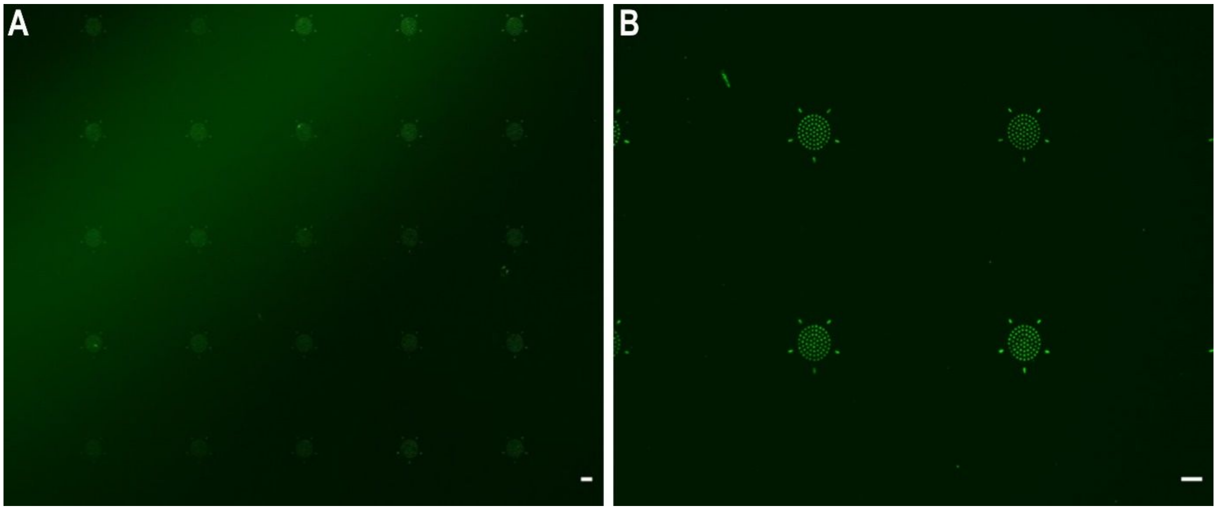

Figure S2. Fluorescent micrograph showing fibronectin immobilization on MHA features. Scale bars: 20 μm.

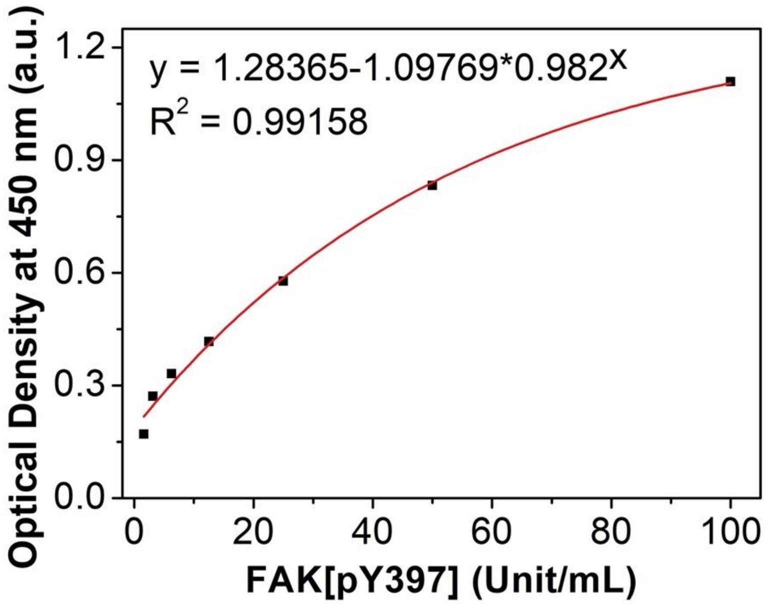

Figure S3. Optical density at 450 nm as a function of focal adhesion phosphorylation kinase concentration fit to a standard curve.

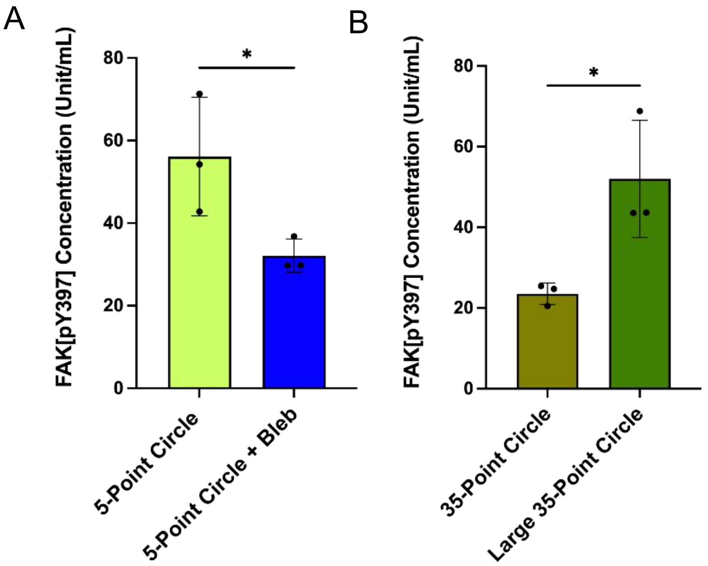

Figure S4. A) Quantitative analysis of focal adhesion dynamics on non-blebbistatin treated 5-point circle patterns vs. blebbistatin-treated 5-point circle patterns using ELISA. Blebbistatin treated patterns exhibited weaker focal adhesions. B) Increasing the aspect ratio of the 35-point circle resulted in enhanced focal adhesion kinase expression. Statistical analysis was performed using an unpaired *t*-test to compare the difference between two groups. *: $p < 0.05$.

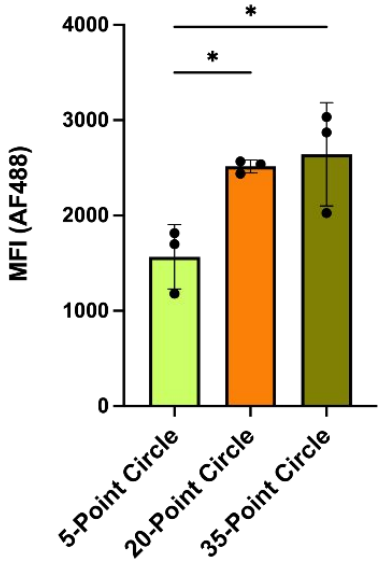

Figure S5. The fluorescence signal of pHrodo green dextran specifically targeting endosomes was also slightly higher in cells seeded on the 35-point circle than on other shapes. Statistical analysis was performed using one-way ANOVA followed by multiple comparison tests using Tukey *post hoc* analysis. *: $p < 0.05$.

Reference

1. Eichelsdoerfer, D. J.; Liao, X.; Cabezas, M. D.; Morris, W.; Radha, B.; Brown, K. A.; Giam, L. R.; Braunschweig, A. B.; Mirkin, C. A. Large-Area Molecular Patterning with Polymer Pen Lithography. *Nat. Protoc.* **2013**, *8*, 2548– 2560.

2. Zhang, W; Meckes, B; Mirkin, C, A; Spherical Nucleic Acids with Tailored and Active Protein Coronae. *ACS. Cent. Sci.* **2019**, *5*, 1983-1990.
